# Gender-Based Variations in Navicular Drop and Foot Morphometry Among North Indian Adults: Implications for Foot Biomechanics and Clinical Assessment

**DOI:** 10.7759/cureus.115325

**Published:** 2026-08-27

**Authors:** Rahul Sharma, Kirandeep Kaur Aulakh, Gitanjali Khorwal, Harpreet Kaur Mohel

**Affiliations:** 1 Anatomy, Maharishi Markandeshwar Institute of Medical Sciences and Research, Ambala, IND; 2 Anatomy, All India Institute of Medical Sciences, Rishikesh, IND

**Keywords:** foot biomechanics, foot drop, foot posture index, navicular drop test, navicular height

## Abstract

Introduction: This study was undertaken to understand the effect of gender on foot morphology, including foot arch and foot index (FI), to comprehend their biomechanical implications and relationships in the North Indian adult population aged 20-50 years.

Methods: A cross-sectional observational study was conducted among 600 healthy participants (300 male and 300 female participants) aged 20-50 years, excluding individuals with lower limb deformities or injuries. Navicular drop (ND) was measured using a standardized technique, including the modified Brody method. The FI was measured as foot breadth divided by foot length, multiplied by 100. Nonparametric statistical tests were applied because the data were nonnormally distributed.

Results: Male participants showed significantly larger foot dimensions (FD; length: 25.40 ± 1.60 cm; breadth: 9.59 ± 0.74 cm) compared with female participants (length: 24.97 ± 1.68 cm; breadth: 9.34 ± 0.93 cm), whereas FI demonstrated no significant difference (p = 0.067). Female participants represent a significantly higher ND (8.29 ± 2.29 mm) than male participants (6.74 ± 2.38 mm), suggesting a decrease in medial longitudinal arch (foot pronation). Though bilateral asymmetry was seen, it was clinically not relevant.

Conclusion: These findings confirm the presence of gender-specific variation in foot morphology, with female participants exhibiting a high ND (foot pronation) and male participants demonstrating larger absolute FDs. The study underscores the importance of incorporating gender considerations into biomechanical assessments, rehabilitation strategies, and orthopedic footwear design, providing valuable evidence to inform personalized clinical and ergonomic interventions.

## Introduction

The human lower limb shows complex biomechanical adaptations that facilitate locomotion, weight-bearing, and dynamic stability [1]. In addition, foot structure is important for the mechanical distribution of carried load and reducing ground reaction forces during walking [2]. Variations in arch structure, such as navicular height (NH), influence lower limb kinematics and are associated with injury risk, thereby increasing their importance in clinical and ergonomic assessments [3]. As a result, the biological essence of the sexual dimorphism of the foot is well known due to the ontogenetic asymmetry in humans [4]. In particular, male participants have large absolute foot-shape factors, whereas a low arch on both sides is specific to female participants [5]. Interestingly, both intrinsic and extrinsic structures can influence the medial longitudinal arch (MLA). However, its function in locomotion is not fully understood [6]. To illustrate, a one-of-a-kind mixed effect pattern is discovered now and then due to modification of NH that appreciably alters lower limb movement [7]. It has recently been recognized that several factors, such as age and physical and other characteristics, affect foot arch. For example, flat feet are more common in older people due to ligament laxity or changes in musculoskeletal structure [8]. Flat feet heighten the risk of injury and discomfort in the foot. Foot arch index or anthropometric measurements, such as the navicular drop (ND) test, are usually investigated to see any change in the foot. Not only age but also gender have been repeatedly identified as determinants of foot arch morphology, as female participants exhibit pronate foot posture and lower arch indices than male participants [9]. Despite increasing evidence, a few studies have been conducted in populations of different ethnic origins. Most of the literature is based on Western datasets, resulting in limited availability of data from other ethnicities, including those in South Asia [10,11]. This lack of literature limits the generalizability of findings and highlights the need for region-specific research. The present study aims to address this gap by examining gender-based variations of foot drop and foot index (FI) within an adult Indian population. The findings help to understand the differences in gender-specific biomechanics and offer empirical data to improve clinical assessments and personalized interventions.

## Materials and methods

Study design

This was a cross-sectional observational study evaluating gender-based variations in ND and FI in 600 adults (300 male and 300 female participants) aged 20-50 years from North India. The study was conducted in the Department of Anatomy, Maharishi Markandeshwar Institute of Medical Sciences and Research, Ambala, Haryana, following approval from the Institutional Ethics Committee (IEC no. 3511). Informed consent was obtained from all participants in both vernacular and English. Participants with a history of lower limb trauma, surgery, musculoskeletal or neurological disorders, or gait abnormalities were excluded.

Measurement procedure

**Figure 1:**
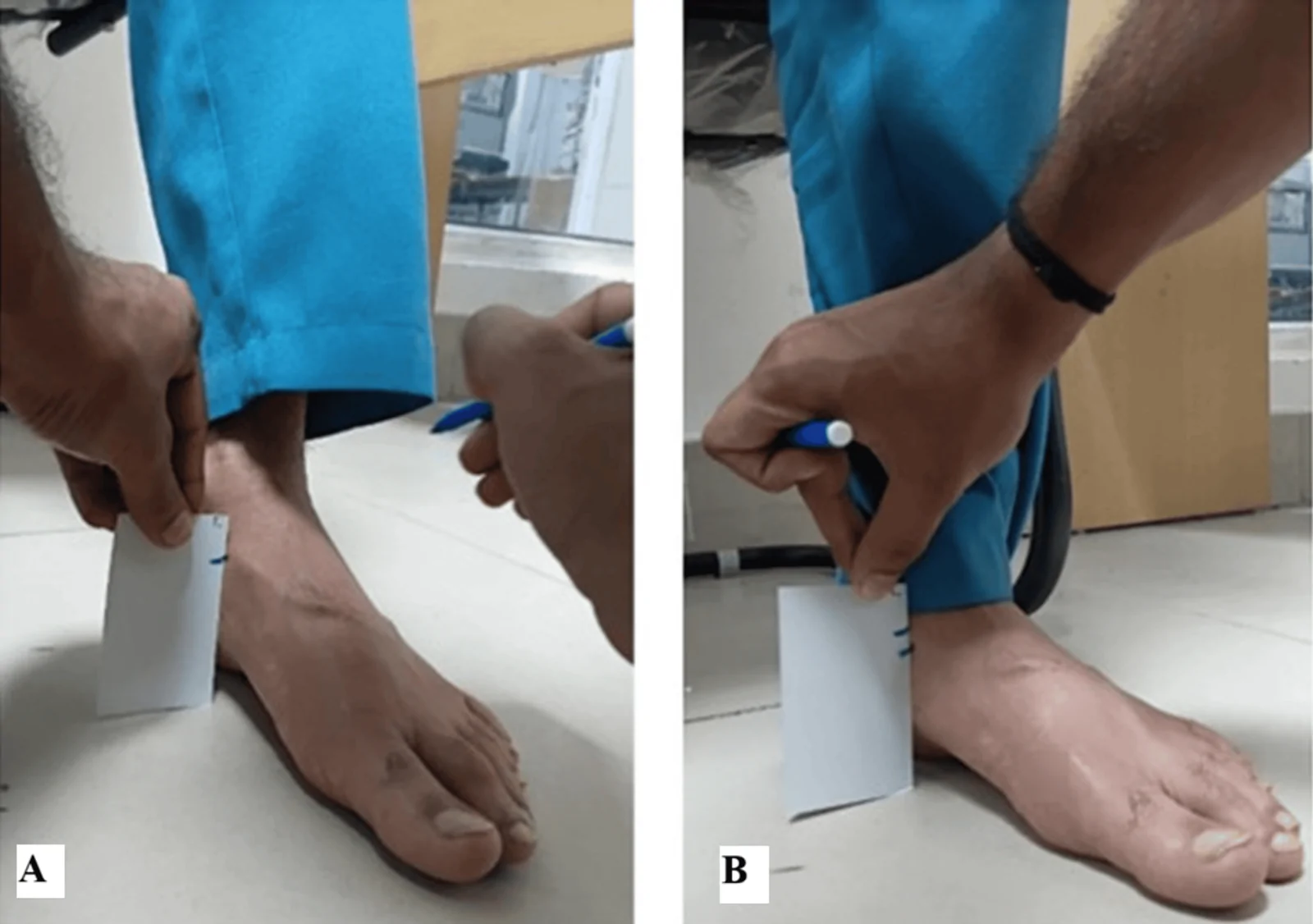
Navicular drop test by using modified Brody technique in (a) non-weight-bearing sitting position and (b) weight-bearing (standing) position

ND and FI measurements were obtained using standardized procedures to ensure reliability and reproducibility. First, the subtalar joint was positioned in neutral; this was achieved by palpating the talar head medially and laterally until a balanced position was attained relative to the navicular bone. ND was assessed using the modified Brody technique, a method known for good intrarater reliability (ICC = 0.89 for the dominant limb and ICC = 0.82 for the non-dominant limb) [12]. The navicular tuberosity was identified by palpation approximately 2.5 cm anterior and inferior to the tip of the medial malleolus and marked with a washable marker for clear visibility. ND was defined as the difference in the height of the navicular tuberosity between the subtalar neutral position (while seated) and the relaxed standing position (Figure 1).

**Figure 2:**
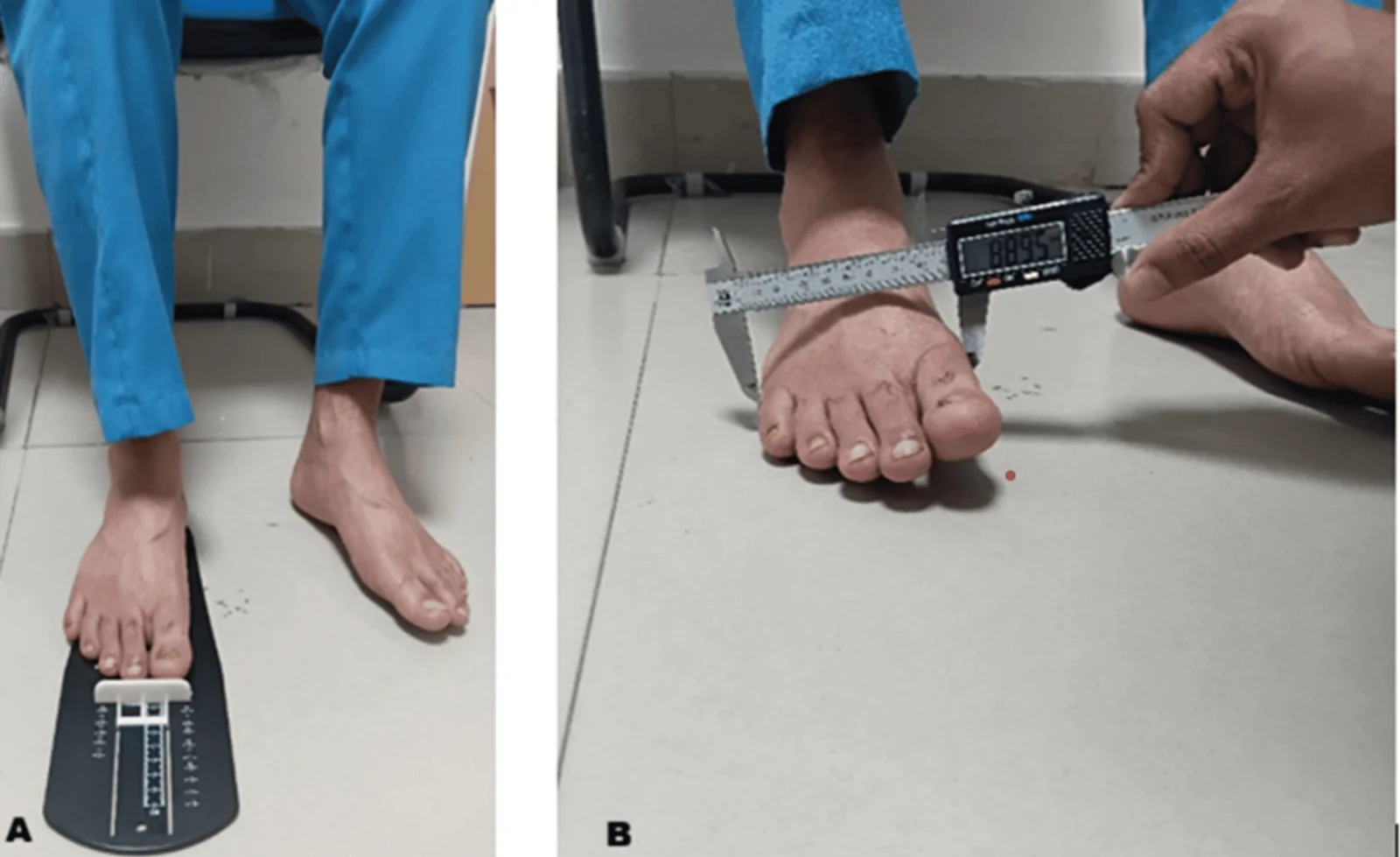
Measurement of (A) foot length by an osteometric foot length measuring board and (B) foot width by a digital vernier caliper in centimeters

Foot length was measured as the linear distance from the pterion (posterior heel) to the acropodion (most anterior point of the longest toe) using an osteometric board (HASTHIP Foot measuring device, Karnataka, India). Foot breadth was defined as the maximum horizontal distance between the first and fifth metatarsal heads and was measured using a digital vernier caliper scaled from 0 to 20 cm and with a marginal error of ±1 mm (Zhart Vernier Caliper Digital 150 mm/6" Video Display measurement tool; Zhart, Shenzhen, China), as shown in Figure 2. The FI was calculated as follows [13]:

$\text{Foot Index} = \left(\frac{\text{Foot breadth}}{\text{Foot length}}\right) \times 100$

Participant height was recorded to the nearest 0.1 cm using a wall-mounted stadiometer (Prime Surgicals height measuring scale; Prime Surgicals, New Delhi, India), with the individual's head positioned in the Frankfurt plane. To minimize bias, all measurements were taken by a single trained investigator, and the mean of three consecutive readings was used to minimize measurement error.

Statistical analysis was performed using IBM Statistical Package for the Social Sciences Statistics software version 27.0.1.0 (IBM Corp., Armonk, NY). Normality of continuous variables was assessed using the Kolmogorov-Smirnov test (p > 0.05). As most variables (ND, foot length, foot breadth) deviated from a normal distribution p < 0.05, nonparametric tests were applied. The Mann-Whitney U test was used to compare variables between male and female groups, while the Wilcoxon signed-rank test was used to assess bilateral (side-to-side) differences within each group. Spearman's correlation coefficient (r) was used to evaluate the relationships between foot drop and foot length, foot breadth, and FI. Statistical significance was set at p < 0.05, and 95% confidence intervals were reported to enhance interpretability and clinical relevance. Effect sizes were calculated to evaluate the magnitude and practical relevance of observed differences.

## Results

This study includes 600 healthy participants, divided equally between 300 male and 300 female participants, with comparable age ranges across both groups. The mean age of male participants was 35.4 ± 9.0 years, and that of female participants was 36.1 ± 8.6 years. Male participants had a mean height of 165.0 ± 8.2 cm, whereas female participants measured 163.7 ± 9.2 cm (p = 0.123). These results show that the study groups were comparable in fundamental demographic traits, indicating that changes in age or stature are unlikely to account for the observed variations in foot morphology (Table 1).

**Table 1:** Mean and standard deviation of age and height of male and female participants SD: standard deviation

Variable	Male (mean ± SD)	Female (mean ± SD)	p value
Age (years)	35.4 ± 9.0	36.1 ± 8.6	0.889
Height (cm)	165 ± 8.2	163.7 ± 9.2	0.123

The comparison of foot morphology and ND between male and female participants shows statistically significant differences in most variables. ND was significantly greater in female participants than in male participants (8.29 ± 2.29 vs. 6.74 ± 2.38 mm, p < 0.001). The point-biserial correlation between gender and ND was moderate (rₚᵦ = 0.31), indicating that female gender was associated with greater ND. This correlation suggests a moderate relationship rather than a causal effect, with gender accounting for approximately 9.6% of the variability in ND.

In contrast, male participants demonstrated significantly greater foot length (25.40 ± 1.60 cm) and foot breadth (9.59 ± 0.74 cm) than female participants (24.97 ± 1.68 and 9.34 ± 0.93 cm, respectively), with both differences statistically significant (p < 0.001) with corresponding effect sizes rₚᵦ = 0.13 for foot length and rₚᵦ = 0.15 for foot breadth. These findings show expected sexual dimorphism in foot dimensions (FDs), with male participants generally having larger absolute FDs and female participants having lower foot arches. However, the FI did not show a statistically significant difference between male (37.88 ± 3.55) and female participants (37.51 ± 4.00) (p = 0.067), with a rₚᵦ value of 0.053 showing little or no association, suggesting that although absolute foot size differs, the comparative relationship between foot length and breadth remains similar across genders (Table 2).

**Table 2:** Comparison between variables of male and female groups performed using the Mann-Whitney U test ^*^p < 0.001 Data are presented as mean ± standard deviation ND: navicular drop

Variables	Male (n = 300)	Female (n = 300)	Test statistics (W)	p value
ND (mm)	6.74 ± 2.38	8.29 ± 2.29	103,210	<0.001^*^
Foot length (cm)	25.40 ± 1.60	24.97 ± 1.68	208,673	<0.001^*^
Foot breadth (cm)	9.59 ± 0.74	9.34 ± 0.93	207,540.5	<0.001^*^
Foot index	37.88 ± 3.55	37.51 ± 4.00	190,976.5	0.067

Although foot morphology was statistically significant (p < 0.001), the magnitude of the differences was small, indicating limited clinical relevance. For example, the mean bilateral difference in foot length (0.43 cm) and in foot breadth (0.25 cm) corresponded to a trivial effect size (r = 0.13-0.15), suggesting that these differences do not show meaningful influence on the functional biomechanics of the foot.

The Wilcoxon signed-rank test revealed a statistically significant difference in ND between the right and left sides in both male and female participants (p < 0.001), indicating bilateral asymmetry of the MLA, with greater variability observed in female participants.

In contrast, foot length, foot breadth, and FI did not show any significant side-to-side differences (p > 0.05), suggesting that foot morphology is structurally symmetrical and independent of side dominance in both genders, as shown in Table 3.

**Table 3:** Bilateral comparison of navicular drop and foot morphometric parameters in male and female participants ^*^p < 0.05, ^**^p < 0.001 SD: standard deviation

Variable	Male			Female		
	Right (mean ± SD)	Left (mean ± SD)	p value	Right (mean ± SD)	Left (mean ± SD)	p value
Navicular drop (mm)	6.73 ± 2.12	6.88 ± 1.83	<0.001^*^	8.08 ± 1.68	8.28 ± 1.83	<0.001^**^
Foot length (cm)	25.8 ± 1.6	25.9 ± 1.7	0.241	24.2 ± 1.5	24.3 ± 1.6	0.284
Foot breadth (cm)	9.6 ± 0.8	9.7 ± 0.9	0.276	9.2 ± 0.8	9.3 ± 0.8	0.312
Foot index	35.6 ± 3.2	35.9 ± 3.4	0.263	36.8 ± 3.5	37.1 ± 3.7	0.298

Spearman’s correlation analysis among male participants shows that ND is positively correlated with foot length (r = 0.11, p = 0.008). However, ND was negatively associated with foot breadth (r = -0.14, p < 0.001) and FI (r = -0.21, p < 0.001), suggesting that higher arches are linked with relatively narrower feet. In female participants, ND shows a positive correlation with foot length (r = 0.02, p = 0.957) and negative correlations with foot breadth (r = -0.09, p = 0.028) and FI (r = -0.09, p = 0.020). These findings suggest that individuals with an increase in ND tend to have larger foot length, with marginally smaller foot breadth and FI. Nevertheless, the correlation coefficients are very close to zero (|ρ| < 0.10) in female participants and (|ρ| = 0.11-0.21) in male participants, indicating small effect sizes and that these relationships are extremely weak and of less clinical significance, despite reaching statistical significance. Therefore, factors such as ligamentous laxity, muscle function, lower limb alignment, and overall foot biomechanics are likely to play a more substantial role in determining ND than FDs alone, as shown in Tables 4, 5.

**Table 4:** Spearman’s correlation matrix of navicular height and foot morphometric parameters in male participants ^*^p < 0.01, ^**^p < 0.001 ND: navicular drop

Correlation	Spearman correlation coefficient (ρ)	p value
ND vs. foot length	0.11	0.008^*^
ND vs. foot breadth	-0.14	<0.001^**^
ND vs. foot index	-0.21	<0.001^**^

**Table 5:** Spearman’s correlation matrix of navicular height and foot morphometric parameters in female participants ^*^p < 0.05 ND: navicular drop

Correlation	Spearman correlation Coefficient (ρ)	P Value
ND vs. foot length	0.02	0.957
ND vs. foot breadth	-0.09	0.028^*^
ND vs. foot index	-0.09	0.020^*^

## Discussion

The present study was conducted to evaluate gender-specific differences in ND and foot morphometry among Indian adults. ND shows a significant difference, with female participants at 8.29 ± 2.29 mm and male participants at 6.74 ± 2.38 mm, whereas FI shows no significant difference between genders. Height measurements for male participants were 165 ± 8.2 cm, compared with 163 ± 9.2 cm for female participants (p = 0.123). These demographic similarities suggest that observed variations in foot morphology are unlikely to be due to age or stature differences. Notably, the height distribution in this Indian cohort was slightly lower than that of the Western population reported in comparable studies [14]. Furthermore, the demographic similarity of the study population allows meaningful comparison with previous studies on gender differences in foot architecture [15].

The ND was significantly higher in female participants (8.29 ± 2.29 mm) than in male participants (6.74 ± 2.38 mm) (p < 0.001). Consequently, gender comparisons are possible with bilateral measures of ND. Previous comparisons have noted side-to-side differences of more than 2-3 mm in some populations [16,17]. Nevertheless, this was not the case here, and this may result from the inclusion of a multiethnic group in previous studies or from variation in physical activity within the study group. In female participants, the ND value was higher, signifying a relatively lower MLA configuration that may affect load transmission throughout the foot during stance and gait. Studies indicate that lower configurations are associated with reduced midfoot mobility and reduced shock-absorption capacity, i.e., greater transmission of ground reaction forces to the proximal joints [18]. Although female participants have smaller feet than male participants, they still exhibit lower navicular arch heights. This may indicate inherent structural differences in the MLA configuration.

Theisen and Day highlighted that the lower extremity functions as a kinetic chain, so changes at one segment can influence the biomechanics of more proximal segments. This kinetic chain relationship provides a biomechanical context for understanding differences in foot posture and lower extremity alignment [19]. Also, Wijnhoven et al. and Boyan et al. noted that gender hormones may be a contributing mechanism underlying the effects of ligamentous laxity and connective tissue properties on arches [20,21]. Similarly, Rosende-Bautista et al. [9] and Soni and Shah [22] said that women have a significantly higher Foot Plantar index, resulting in lower arch height than normal in male participants. Contrary to the study, Nielsen et al. state that ND is not influenced by age and body mass index (BMI) [10].

This study also shows that foot length (25.40 ± 1.60 cm) and foot breadth (9.59 ± 0.74 cm) are significantly greater in male participants than in female participants (24.97 ± 1.68 cm; 9.34 ± 0.93 cm) (p < 0.001); foot structure asymmetry is affected by limb dominance, as per an earlier study [16]. However, the differences in magnitude were very small for foot length (0.43 cm) and foot breadth (0.25 cm), corresponding to trivial effect sizes (r = 0.13-0.15). This suggests that there will be no significant bias when comparing right- and left-side foot length and breadth across both genders. Therefore, these differences should be interpreted as evidence of gender-related variation in absolute FDs rather than as clinically important functional differences. Their main practical relevance concerns footwear fit, anthropometric classification, and ergonomic/orthotic design rather than the direct impairment of foot function.

The FI in male participants (37.88 ± 3.55) and in female participants (37.51 ± 4.00) does not differ significantly (p = 0.067). According to a study by Sharma et al. involving the Jat and Baniya communities of North India, the researchers found that foot length and foot breadth showed significant differences between the populations, whereas the FI showed no significant difference (p < 0.001) [13]. This contrasts with previous reports stating that FI is significantly greater in male participants than in female participants [23]. Ethnic differences could account for variations in foot proportions or in parameter measurements. This means that the absolute sizes of the feet differ between the two genders, but the geometric ratios that define their shape remain stable.

The above finding has relevant clinical applications for prescribing orthotics to people or athletes who frequently utilize special footwear. The differences between genders in foot arch necessitate designing an arch support according to gender. Based on shoe sizes, tapping, and other cast measurements, differences in scale are sufficient to design gender-specific patterns [24]. Zhai et al. describe that orthotic insoles or foot arch support help to support the flat and high arch foot condition and prevent foot injury and plantar fasciitis in the long run [25]. Similarly, Wang et al. found that kinematic taping for flexible flat feet immediately reduces abnormally elevated foot pressure, tone, and stiffness by supporting the lower extremity muscles [26]. Kinesiology tape is usually used to provide support by maintaining an artificial arch in the foot.

Future studies should be carried out to assess the functional relevance of gender differences under dynamic conditions. Comprehensive gait analyses are needed to evaluate how male and female foot morphologies influence kinetic and kinematic parameters across various activities.

Furthermore, investigating the relationship between foot arch variability and injury susceptibility may provide valuable insights for preventive strategies. Longitudinal studies are also required to explore the developmental trajectory of these two differences between FD and FI, whether they arise during puberty or continue consistently across the lifespan, and their association with gender specific injury patterns such as stress fractures and plantar fasciitis. Advanced imaging modalities, including weight-bearing MRI, could further clarify the contributions of soft tissues to arch morphology.

Limitation

This study is limited by its dependence on static measurements, which may not fully capture dynamic foot function during gait. The absence of BMI and activity-level data represents a potential confounder, as both factors are known to influence foot morphology. Additionally, the cross-sectional design precludes causal inference regarding the developmental origins of observed gender differences.

## Conclusions

This study provides robust evidence of gender-specific variations in foot morphology within an adult Indian population. Female participants exhibited significantly lower navicular arches, whereas male participants showed larger absolute FDs, despite comparable FI values between genders. Incorporating gender-specific insights into orthotic design, rehabilitation strategies, and ergonomic standards has the potential to enhance clinical effectiveness and reduce injury risk.
